# A Decade of Exploring the Mammalian Sperm Epigenome: Paternal Epigenetic and Transgenerational Inheritance

**DOI:** 10.3389/fcell.2018.00050

**Published:** 2018-05-15

**Authors:** Alexandre Champroux, Julie Cocquet, Joëlle Henry-Berger, Joël R. Drevet, Ayhan Kocer

**Affiliations:** ^1^GReD, Laboratoire “Génétique, Reproduction and Développement,” UMR Centre National de la Recherche Scientifique 6293, INSERM U1103, Université Clermont Auvergne, Clermont-Ferrand, France; ^2^INSERM U1016, Institut Cochin, Centre National de la Recherche Scientifique UMR8104, Université Paris Descartes, Sorbonne Paris Cité, Paris, France

**Keywords:** sperm, paternal epigenome, chromatin, histone post-translational modifications, protamines, embryo development, DNA methylation, sperm non-coding RNA

## Abstract

The past decade has seen a tremendous increase in interest and progress in the field of sperm epigenetics. Studies have shown that chromatin regulation during male germline development is multiple and complex, and that the spermatozoon possesses a unique epigenome. Its DNA methylation profile, DNA-associated proteins, nucleo-protamine distribution pattern and non-coding RNA set up a unique epigenetic landscape which is delivered, along with its haploid genome, to the oocyte upon fertilization, and therefore can contribute to embryogenesis and to the offspring health. An emerging body of compelling data demonstrates that environmental exposures and paternal lifestyle can change the sperm epigenome and, consequently, may affect both the embryonic developmental program and the health of future generations. This short review will attempt to provide an overview of what is currently known about sperm epigenome and the existence of transgenerational epigenetic inheritance of paternally acquired traits that may contribute to the offspring phenotype.

## Introduction

**Figure 1:**
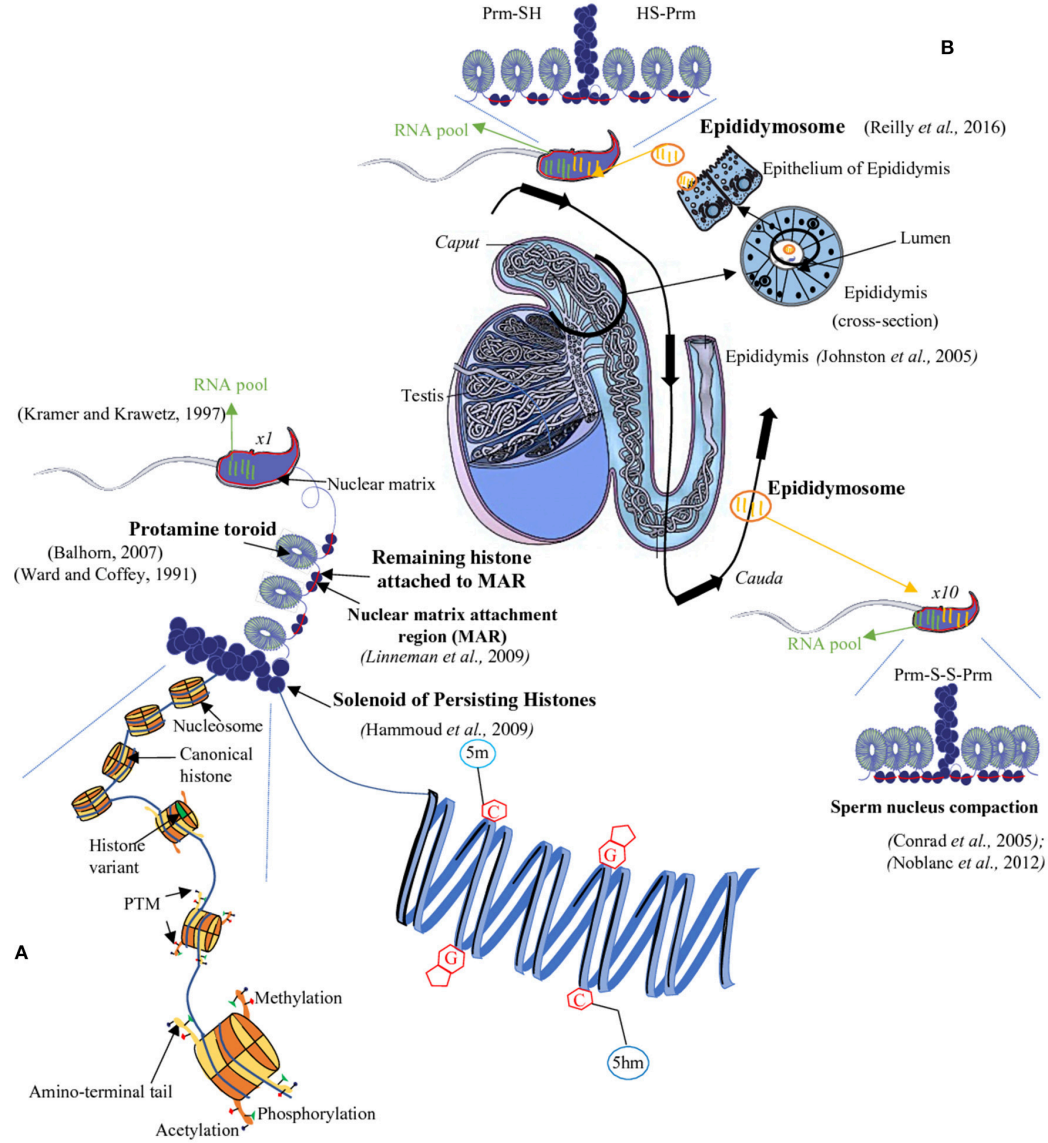
The sperm epigenome. During spermatogenesis, most histones are replaced by protamines (PRM). However, few histones remain in chromosomal domains called ≪ solenoid ≫ as well as in the short DNA segments connecting two adjacent toroids which are also attached to the sperm nuclear matrix (Matrix Attachment Regions = MAR, red bar). The sperm DNA is thus mainly packaged into toroids (protamine rich regions) and, for a minor proportion, into solenoids (histone rich regions) overall allowing a great condensation of the chromatin **(A)**. This condensed state of the sperm nucleus is further enhanced during epididymal maturation *via* intra- and inter-protamine disulfide bonds **(B)**. The sperm nucleus also harbors epigenetic marks at various levels: for example, on the sperm DNA there are complex methylation profiles with regions rich in 5-methylcytosine (5 mC) as well as in 5-hydroxymethylcytosine (5hmC). At the level of the sperm chromatin, persisting histones are concerned by a vast array of post-translational modifications (PTM; **A**). Finally, a third epigenetic information is associated with the sperm cell it is represented by a complex pool of RNA (mRNA and several classes of non-coding RNA). This RNA-mediated epigenetic information is acquired both during spermatogenesis and the post-testicular maturation processes (i.e., during epididymal transit; black arrow) with non-coding RNA being transferred from the epididymal epithelium toward sperm cell *via* lipid-rich exosomes named, epididymosomes **(B)**. C, cytosine; G, guanine; SH, thiol group; S-S, disulfide bridge.

The principal function of spermatozoa is to deliver the haploid paternal genome to an oocyte during fertilization. Spermatozoa are highly specialized cells generated in the testis through a differentiation process called spermatogenesis. They are equipped with specific structures to achieve fertilization, such as a flagellum which confers mobility and a very compact nucleus which ensures protection of the paternal genome. Their chromatin organization differs significantly from that of somatic cells: while, in the latter, the chromatin is packed into nucleosomes containing 146 bp of DNA wrapped around an octamer of basic proteins named histones (Kornberg, 1974), in mammalian sperm cells, the chromatin is organized in a very compact structure described as a “doughnut loop” or “toroid” in which the DNA is wrapped around even more basic, smaller proteins, called protamines. As a result, the sperm nucleus is about seven times smaller than that of an interphase somatic cell (Ward and Coffey, 1991). During the last step of spermatogenesis, histones are progressively replaced by protamines through a series of complex chromatin remodeling events which have been the focus of recent studies (see for instance, Gaucher et al., 2012; Montellier et al., 2013; Barral et al., 2017; Liu et al., 2017). The key steps of histone-to-protamine transitions are (i) opening of the chromatin, owing to the combination of post translational modifications of histones, in particular histone hyperacetylation, and incorporation of histone variants, (ii) eviction of most histones while transition proteins and some late expressed histone variants are transiently incorporated, (iii) removal of transition proteins and of most remaining histones, while protamines are incorporated (For reviews on this topic, see Rathke et al., 2014; Hoghoughi et al., 2017). The majority of histones are replaced by protamines during spermatogenesis but some do persist in mature sperm (Figure 1; Tanphaichitr et al., 1978; Gatewood et al., 1990; Oliva and Dixon, 1991; Wykes and Krawetz, 2003). First seen as histone remnants of an inefficient replacement process, it is now rather clear to the scientific community that sperm persisting histones contribute to the paternal information brought to the oocyte and the developing embryo. There is nevertheless an ongoing debate regarding the genomic location of those persistent histones (Hammoud et al., 2009; Brykczynska et al., 2010; Carone et al., 2014; Samans et al., 2014; Kocer et al., 2015; Royo et al., 2016). Interestingly, it has also been recently demonstrated that spermatozoa do not only transmit their DNA and chromatin to the embryo. Along with the haploid genome and various epigenetic marks carried by the DNA and associated chromatin proteins, spermatozoa bring along a complex array of RNAs (both coding and non-coding). Recent reports have shown that some of these RNAs may affect the developing embryo and/or the progeny (Chen et al., 2016a; Sharma et al., 2016).

Therefore, from now on, the issues of male fertility, successful reproduction, optimal embryonic development, offspring's health and that of the subsequent generations cannot be solely attributed to sperm DNA integrity. The picture is now more complex and beside an optimal paternal genetic code one has to consider the highly dynamic and environmentally susceptible epigenetic information carried by the paternal nucleus.

In the present review, we focus on sperm-specific chromatin features and epigenome. Subsequently, with the literature available to date we illustrate the impact that the sperm epigenome may have on early embryo development and the consequences of environmental effects on paternal epigenome inheritance.

## The mammalian sperm epigenome

Epigenetics (from “epi” in Greek which means above/upon/near) is the study of heritable changes in gene expression that are not caused by modifications in the primary DNA sequence (Goldberg et al., 2007). Epigenetic information is cell specific, dynamic and responsive to environmental influences. Epigenetic regulation can occur *via* at least three main processes in mammals: (i) DNA methylation and associated modifications, (ii) the histone/chromatin code which consists mainly in histone variants and their post-translational modifications, and (iii) Coding and non-coding RNA.

## DNA methylation and beyond

DNA methylation is the most studied epigenetic modification in the literature, probably owing to its robustness compared to histone post-translational modification (PTM) or RNA.

DNA methylation is an important regulator of gene expression demonstrated to be globally involved in gene regulation, and, more specifically, in transposon silencing, genomic imprinting, maintenance of genome integrity or X chromosome inactivation (for reviews, see Bird, 2002; Hackett et al., 2012; Messerschmidt et al., 2014). It consists in the addition of a methyl group of S-adenosyl-1-methionine to carbon five of cytosine resulting in the formation of 5-methylcytosine (5mC). In mammals, cytosine methylation occurs mostly in a context of CpG dinucleotides. CpG islands (CGIs), which possess high CpG density (i.e., ~1 kb-long regions with greater than 50% CpG), are predominantly unmethylated. Other CpGs (in a non-CGI context) are usually methylated (Deaton and Bird, 2011; Messerschmidt et al., 2014). Overall, DNA methylation at promoter regions is associated with gene repression (except for promoters with low CpG content, for review see Messerschmidt et al., 2014). DNA methylation is carried out by DNA methyltransferases (DNMTs) that are divided in two classes: DNMT3A, DNMT3B, and DNMT3L (Bestor T., 1988; Bestor T. H., 1988) are responsible for *de novo* methylation, while DNMT1 is involved in methylation maintenance (for review, see Chen and Li, 2004). A fourth de novo DNMT has been recently identified in rodents (Barau et al., 2016; Jain et al., 2017).

In gametes, DNMTs also establish a differential DNA methylation on ICR (Imprinting Control Regions) a phenomenon known as paternal or maternal imprinting depending on whether it takes place in male or female germ cells (Kaneda et al., 2004; Kato et al., 2007). These ICR are maintained in the embryo meaning that somatic cells present, at a few loci, paternal or maternal mono-allelic methylation patterns which results in a maternal or paternal mono-allelic expression, respectively (Hanna and Kelsey, 2014). This phenomenon only concerns a minor part of the genome (to date there are fewer than 30 ICR in the mouse genome) but has an important signification in term of development and health. Most of them are maternal ICR and only 3 of them are established in male gametes.

Transposon silencing is also mediated by DNMTs and established in the male gametes with a high efficiency.

All these distinct sex-dependent DNA methylation profiles are essential for male gametogenesis as demonstrated by reports that loss of function of DNMT3A and 3B leads to an arrest in meiosis, an overexpression of repeated elements such as LINEs (long interspersed nuclear elements) and IAP (intracisternal A particles) and, a loss in spermatocytes *via* apoptosis; while loss of function of DNMT3L leads to male sterility because *de novo* methylation is absent in germ cells (Walsh et al., 1998; Bourc'his and Bestor, 2004; Kaneda et al., 2004; Kato et al., 2007). In human, a recent study shows that single-nucleotide polymorphisms (SNPs) in different *DNMT* genes induce idiopathic male infertility associated with abnormal semen parameters (Tang et al., 2017).

Overall, depending on the cellular type and development period, the genome is more or less methylated with two major reprogramming time points during mammalian development: in primordial germ cells (PGC) and pre-implantation embryos.

During the formation and migration of PGC at E12.5 to E13.5 days in mouse, DNA methylation is erased up to a point that solely 10% of total CpG remain methylated. This is required because PGC are derived from embryo cells that have already acquired a somatic fate (Hajkova et al., 2002; Guibert et al., 2012; Seisenberger et al., 2012). The erasure of CpG methylation (5mC) is a key component of PGC specification, but the dynamics and underlying mechanisms of this process remained unclear (Hackett et al., 2012). Recent studies have shown that conversion of 5mC to 5hmC (5-hydroxymethylcytosine) is instrumental to the DNA demethylation process and involves enzymes belonging to the ten-eleven translocation (Tet) family (Tahiliani et al., 2009; Gan et al., 2013; Hackett et al., 2013). Further down, 5hmC can itself be oxidized by Tet into 5fC (standing for 5-formylcytosine) and 5caC (standing for 5-carboxycytosine; He et al., 2011; Ito et al., 2011). In mammals, the Tet family contains three members (Tet 1–3) that are expressed differently according to tissue and stage of development (Tahiliani et al., 2009). Tet 1 and 2 are present in embryonic stem (ES) cells and PGC (Hackett et al., 2012) while Tet3 is expressed in the oocyte, spermatozoon and in the preimplantation embryo. In mouse, PGC, at around E15.5, DNA methylation is rapidly re-acquired, and the germ cell-specific profile is fully established after birth in pachytene spermatocytes through the action of DNMT 3A, 3B, 3L, and 3C (Guibert et al., 2012; Seisenberger et al., 2012; Barau et al., 2016).

At the end of gametogenesis, spermatozoa are extensively methylated with ~90% of methylated CpG, a situation that is different from that of the oocyte where only 40% at CpG are methylated (Popp et al., 2010). DNA modifications in spermatozoa are mostly in transposons and intergenic regions, while gene bodies and CGI are sparsely methylated (Smith et al., 2012). In many species, the sperm DNA methylation pattern appears to be different from that of somatic cells but similar to that of ES cells (Weber and Schübeler, 2007; Farthing et al., 2008). For instance promoters of transcription and signaling factors controlling early development such as *Sox-, Fox-, Hox or Gata*- family are equally hypomethylated in ES cells and spermatozoa (Hammoud et al., 2014). Interestingly, these same regions apparently contain persisting histones (Hammoud et al., 2009; Brykczynska et al., 2010; Erkek et al., 2013) suggesting a role of the sperm epigenome in the regulation of gene expression after fertilization.

In the oocyte, zygote and early embryo, the DNA methylation pattern is the opposite, *i.e*. methylation restricted to CGI and gene bodies (Smith et al., 2012). Other cytosine modifications, such as 5hmC, 5fC, and 5caC are minor compared to 5mC but despite representing only a small fraction of modified CpG, their location could be biologically meaningful. In their study of 5hmC at different stages of male germ cell differentiation, Gan et al. observed that 5hmC represents less than 2.5% of 5mC in male germ cells but is very dynamic as it changes in genomic regions related to the regulation of gene expression (such as transposons and piRNA clusters) and correlates positively with genes expressed during spermatogenesis, with high level of 5hmC found in coding exons of highly expressed genes (Gan et al., 2013).

In mouse, a few days after fertilization, DNA methylation is erased, with substantial differences in timing between the paternal and maternal pronuclei (Messerschmidt et al., 2014). However, a small portion of DNA methylation, principally imprinted regions and a sub-category of transposons called IAP (Intracisternal A-particle) is resistant to this genome-wide reprogramming with potential for transgenerational inheritance (see below).

Numerous studies have correlated abnormal sperm DNA modification patterns with male infertility (for review, see Cui et al., 2016), starting with defects in DNA methylation on ICR that are associated with failures in spermatogenesis (Hartmann et al., 2006; Denomme et al., 2017). In clinic, patients showing overall low DNA methylation or a non-methylated *Igf2/H19* locus present a reduced sperm quality, a decrease in sperm count and mobility when compared to fertile men (Niemitz and Feinberg, 2004; Boissonnas et al., 2010; Zama and Uzumcu, 2010). Other abnormal profiles in DNA methylation on imprinting gene regions such as *Mest* (mesoderm specific transcript) or on spermatogenesis implicated genes such as for example *Dazl* (deleted in azoospermia like) were shown to be associated with oligozoospermia, i.e. low sperm concentration (Marques et al., 2008; Poplinski et al., 2010). More recently, it was also reported that modification in 5hmC pattern in sperm is associated with male infertility (Wang et al., 2015; Efimova et al., 2017). In these reports, infertile males were shown to contain higher rate of 5 hmC than fertile males and infertility correlated with defects in sperm morphology and a high sperm DNA fragmentation rate (Efimova et al., 2017). Several studies have also reported that a link exists between assisted reproductive technologies (ART) and loss of imprinting resulting in an increased incidence of genomic imprinting disorders in children conceived *via* ART (for review, see Ventura-Juncá et al., 2015). These include Beckwith-Wiedemann syndrome (Chang et al., 2005), Angelman syndrome, Silver-Russel syndrome (Chopra et al., 2010) and retinoblastoma (Marees et al., 2009).

Nevertheless, a study shows that mice produced by intracytoplasmic sperm injection (ICSI) present altered DNA methylation on imprinted genes (de Waal et al., 2012) but offspring of ICSI-derived males exhibited normal epigenetic profiles in their somatic tissues, suggesting correction of the observed altered epigenome by germ-line specific epigenetic reprogramming (de Waal et al., 2012). These results suggested that ART procedures can lead to epigenome alterations that are normally corrected in the germ line through epigenetic reprogramming and thus not propagated to subsequent generations.

## Sperm chromatin

The eukaryotic genome is compacted into the nucleus *via* a multi-layer structure, the chromatin. The association of a histone octamer with ~146 bp of DNA forms the basal chromatin structure of all somatic cells, the nucleosome. The organization of the sperm chromatin is unique compared to that of other cells, as its basal unit is the nucleoprotamine. During the last phase of spermatogenesis takes place an extensive remodeling of male germ chromatin that results in the replacement of most histones by smaller and more basic proteins than histones, the protamines. This leads to a chromatin structure known as “doughnut loop” or “toroid” containing 50–100 Kb of DNA (depending on the species analyzed). This structure is ~6–10 times more condensed than a classical somatic nucleosome (Ward and Coffey, 1989, 1991; Balhorn, 2007). In mammals, including human, two protamines, Prm1 and Prm2 exist. The ratio between the two Prm is 1:1 in human sperm but shows a wide range of variability between species (Corzett et al., 2002). However, the protamine ratio is tightly controlled and an aberrant ratio is associated with male infertility (Balhorn et al., 1988; Aoki et al., 2006). In order to study the structure and composition of the mammalian sperm chromatin, two main approaches have been used: either DNA digestion by endonucleases, such as DNase I or Mnase (Pittoggi et al., 1999; Zalenskaya et al., 2000; Hammoud et al., 2009; Brykczynska et al., 2010; Erkek et al., 2013), or high salt treatments to disrupt proteins-DNA associations coupled to digestion of free DNA typically by using EcoRI and BamHI endonucleases (Wykes and Krawetz, 2003; Arpanahi et al., 2009). Taken together, these studies showed that the sperm DNA is mainly associated with protamines and that only few regions remain associated with histones both canonical and variant ones (Hammoud et al., 2009; Brykczynska et al., 2010; Erkek et al., 2013). From these studies, it was found that about 2% histones persist in the sperm nucleus of mouse, hamster and bull, while in human around 5–10% of histones remain (Balhorn et al., 1977; Gatewood et al., 1990; Bench et al., 1996; Tovich and Oko, 2003; Hammoud et al., 2009; Erkek et al., 2013). Persisting histones were reported to be organized into solenoid structures within the protamine embedded chromatin and also, associated with linker DNA segments connecting adjacent toroids, a chromatin structure formed by protamines (Sotolongo et al., 2003; Ward, 2010; Noblanc et al., 2013) as schematized in Figure 1.

Chromatin organization in somatic cells depends on the composition of nucleosomes in histone variants and histone PTMs. To date, the most extensively studied histone PTMs are lysine acetylation (which often correlates with nucleosome destabilization and transcriptional activity) and methylation, which depending on the residue can impact on gene expression either by activation or repression. For example, histone H3 trimethylated at lysine 27 (termed H3K27me3) deposited by the histone-lysine N-methyltransferase enzyme EZH2, leads to chromatin compaction and thus to inhibition of transcription. This mark is notably found in the constitutive heterochromatin. In contrast, H3K4me3 (i.e., trimethylation at lysine 4 of histone 3) is associated with actively transcribed genes (Barski et al., 2007; for review, see Bernstein et al., 2007). The recent development of mass spectrometry-based analyses of histone PTM has immensely complicated the picture with 20 different types of modifications identified to date resulting in several hundred different histone PTMs (for review, see Huang et al., 2015; Andrews et al., 2016). Also, a recent study identified 11 PTMs on protamines both in mouse and human sperm including phosphorylation and acetylation (Brunner et al., 2014). The authors suggested that PTMs of protamines are involved in the process of deposition of protamines in sperm and their eviction after fertilization. These concurred with earlier observations that phosphorylation of Prm was necessary for its deposition on DNA during spermatogenesis (Dadoune, 2003).

Gene expression and chromatin organization in somatic cells is also influenced by nucleosome remodeling, i.e. incorporation of histone variants that have different properties and functions compared to that of canonical histones leading to subtle changes in chromatin organization. The incorporation of histone variants depends on cellular types and cell cycle (for review, see Talbert and Henikoff, 2017).

Histone PTMs and histone variants also play a major role in the reorganization of sperm chromatin. First, because they are involved in the extensive chromatin remodeling process that takes place in elongating spermatids and leads to the replacement of histones by protamines. Secondly, because a small portion of histones (~1–10% depending on species) is maintained in spermatozoa, and those persisting histones bear PTMs and include non-canonical histones (so called histone “variants”). It is worth noting that a majority of histone variants are encountered at one point during spermatogenesis; they are involved in establishing a male germ cell-specific gene expression program and/or in histone-to-protamine chromatin remodeling process (see Table 1). Consequently, deficiency in histone variants is often associated with defective spermatogenesis (see Table 1).

**Table 1:** Variant histones in mammalian sperm nucleus.

**Acronym**	**Description**	**Phenotype of knockout mice**		**References**
H1t	Testis-specific variant of histone H1	No data		Drabent et al., 2003
H1t2	H1 histone family, member N, testis-specific (H1fnt)	Male infertility due to sperm elongation and nucleus condensation defects		Martianov et al., 2005; Tanaka et al., 2005
γH2A.X	Specifically present at DNA breaks during sperm chromatin remodeling	Male infertility due to failure of sex-body formation and defects in pairing meiotic sex chromosome in spermatocytes		Celeste et al., 2002; Fernandez-Capetillo et al., 2003; Blanco-Rodríguez, 2009; Turinetto and Giachino, 2015
H2A.Z	Involved in transcription, DNA repair, chromatin cohesion, centromeres structures and eu/heterochromatin boudaries maintainance	Early embryo lethality		Suto et al., 2000; Meneghini et al., 2003; Greaves et al., 2007; Xu et al., 2012; Sharma et al., 2013
MacroH2A Or mH2A	Associated to heterochromatin and interaction with histones deacetylases, silencing of sex chromosomes during spermatogenesis	Reduced litter Perinatal death in the absence of two MacroH2A isoforms		Pehrson and Fried, 1992; Pehrson et al., 2014
TH2A	Testis-specific variant of histone H2A	No data	When both genes are knocked-out: male infertility due to male infertility due to sperm nucleus condensation defects	Trostle-Weige et al., 1982
TH2B	Testis-specific variant of histone H2B	No phenotype Canonical H2B compensation		Montellier et al., 2013; Shinagawa et al., 2015
H2A.L1 & 2	Found in condensed spermatids on pericentromeric heterochromatin	Male infertility associated with sperm chromatin decondensation		Govin et al., 2007; Barral et al., 2017
H3.3	Variant of histone H3	Male infertility due to sperm chromatin decondensation associated lack of Prm protein		Szenker et al., 2011; Yuen et al., 2014
H3t	Testis-specific variant of histone H3	Male infertility associated with loss of spermatocytes		Govin et al., 2004; Ueda et al., 2017
H3.5	Testis-specific variant of histone H3 in human	No data		Schenk et al., 2011
CENP-A	Centromeres-specific variant of histone H3	Embryos die at preimplantation stage		Palmer et al., 1991

*Beside the names (acronyms and extended names) of the histone variants found in the mouse sperm nucleus, the table reports the effects recorded when the corresponding gene was knocked-out. Prm, Protamine*.

Prior to histone eviction, in spermatids, histone variants are incorporated and many histone residues, modified. This leads to nucleosome destabilization and chromatin opening (for reviews, see Rathke et al., 2014; Hoghoughi et al., 2017). Histones are replaced by a transitory structure composed of transition proteins (TPs) that regulate protamine processing and assembly (Goudarzi et al., 2014). Interestingly, one particular spermatid-expressed histone variant H2A.L2 is incorporated along with transition proteins after most histones have been removed, and facilitate nucleoprotamine assembly (Barral et al., 2017). In addition, many modifications of histone residues have been reported to occur prior to histone removal; the most striking and described in the literature being histone H4 hyperacetylation (see Goudarzi et al., 2014). It is the molecular signal recognized by the testis-specific bromodomain protein BRDT which controls the replacement of histones by transition proteins (Gaucher et al., 2012). Other chromatin remodeling proteins and enzymes have been described to be involved in histone to protamine transition. This is the case of CHD5 (Chromodomain helicase DNA binding protein 5; Li et al., 2014; Zhuang et al., 2014), CDYL (Chromodomain Y-like transcription corepressor) which acts as a crotonyl-coA-hydratase (Liu et al., 2017), the histone acetyl transferase TIP60 (Dong et al., 2017), the ubiquitin ligase RNF8 (Lu et al., 2010) or PIWI (Gou et al., 2017). In human, low germ cell expression of CHD5 and BRDT is associated with male infertility (Steilmann et al., 2010; Li et al., 2014; Zhuang et al., 2014).

Along with histone H4 acetylation at multiple sites, other PTMs occur on histones prior to their turn-over, such as methylation, phosphorylation, crotonylation and ubiquitinylation of histone H3 and H2B residues (Wendt and Shilatifard, 2006; Govin et al., 2010; Lu et al., 2010; Tan et al., 2011; Brunner et al., 2014; Dottermusch-Heidel et al., 2014a,b; Pentakota et al., 2014; Rathke et al., 2014; Mishra et al., 2015; Hada et al., 2017; Hoghoughi et al., 2017). Some of them are transmitted to the embryo *via* the persisting histones; their role and effect remain to be determined.

Finally, chromatin organization and gene regulation also depend on a higher order (3D) chromatin structure, in which megabase-size genomic regions form topologically associated domains (TADs; Dixon et al., 2012; Sexton et al., 2012). Those domains of higher order chromatin structure are stable during cell differentiation and conserved through evolution (Dixon et al., 2015). They can be partitioned into different compartments (A/B compartments; Lieberman-Aiden et al., 2009) which present good correlation with active and repressive chromatin regions, as defined by the composition in histone PTMs. Quite surprisingly in view of the different architectures of sperm and somatic cell chromatin, somatic TADs appear to be partially conserved in spermatozoa. High resolution maps nevertheless showed an elevated number of additional long-range intra-chromosomal interactions (>2 Mb) many of which occurring between different TADs (inter-TADs), as well as inter chromosomal contacts (Battulin et al., 2015; Jung et al., 2017; Ke et al., 2017). Interestingly, the organization in A/B TADs compartments in sperm correlates with the presence of persisting histones. B compartment are “repressive” domains (genes in B compartments showing lower expression than those in A compartments) that are enriched in persisting histones (Ke et al., 2017), as measured by Micrococcal nuclease (Mnase) digestion assays (see below).

A burning question related to the sperm epigenome is the location, function and contribution to the embryo of the small portion of histones that persist in spermatozoa. To address this question, several groups have undertaken studies consisting in Mnase digestion of sperm chromatin followed by high throughput sequencing (Hammoud et al., 2009; Brykczynska et al., 2010; Erkek et al., 2013; Carone et al., 2014; Samans et al., 2014), but the answer is not clear to date. While it is clear that the positioning of sperm remaining histones is not random, there is an ongoing debate about the location in the sperm genome of these histones (Arpanahi et al., 2009; Hammoud et al., 2009, 2011; Brykczynska et al., 2010; Erkek et al., 2013; Noblanc et al., 2013; Carone et al., 2014; Samans et al., 2014; Royo et al., 2016). Some studies have shown that persisting histones are enriched at promoters of genes involved in early embryonic development (e.g., transcription factors, *HOX* genes, signaling proteins, etc.), microRNAs clusters, genes subjected to genomic imprinting and binding sites of the chromatin insulator protein CCCTC-binding factor (CTCF; Arpanahi et al., 2009; Hammoud et al., 2009). Besides, nucleosomes were also found to be preferentially retained at sparse methylated DNA sequences enriched in CpG (Hammoud et al., 2011; Erkek et al., 2013). When focusing on histone PTMs, those groups observed enrichment of H3K4me2/3 and H3K27me3 bivalent marks at promoters of developmental genes, while H3K4me2/3 alone was found at the promoter of genes with a role in spermatogenesis and H3K27me3 alone marks promoters of genes repressed during gametogenesis and early embryo development (Hammoud et al., 2009; Brykczynska et al., 2010). However, histone variant TH2B was found in gene promoters involved in sperm maturation, fertilization and capacitation while H2A.Z was detected at peri-centromeric regions (Greaves et al., 2006; Hammoud et al., 2009). These data suggest that the organization of the paternal genome could influence early embryonic development (also see below). Other reports have, however, found that persisting histones are predominantly associated with intergenic sequences outside of gene regulatory regions (Carone et al., 2014; Samans et al., 2014; Kocer et al., 2015). Carone et al. (2014) reported that nucleosomes are mainly distributed in gene-poor regions in the mouse sperm, and that a subcategory of nucleosomes is retained at CTCF binding sites (Carone et al., 2014). Samans et al., showed nucleosome enrichment within distal intergenic regions and introns as well as with centromere repeats and retrotransposons including LINE1 and SINEs, (Samans et al., 2014). But the computational approach used in this study was contested as it appeared to induce a bias toward repetitive elements (Royo et al., 2016). These rather conflicting data may be also partly explained by the different protocols that were used in order to recover histone-bound sperm DNA domains (Kocer et al., 2015). In fine, to date, there is no strong consensus as to which specific DNA sequences in sperm are associated with nucleosomes.

## Sperm RNA

Another category of epigenetic regulators of gene expression is the vast and heterogeneous family of non-coding RNAs (ncRNA). They can be grouped according to their size in long or small non-coding RNA. In this review, we will focus on small non-coding RNA (sncRNA) as they are the most described in male germ cells. In spermatozoa, sncRNA could be the basis of epigenetic information transmitted to the embryo as they enter the oocyte upon fertilization. At the end of spermatogenesis, most of the cytoplasm and RNA content of spermatozoa are ejected. Only a limited number of RNA molecules remain, and they have been the focus of several studies. In addition to mRNA fragments, several small non-coding RNA (sncRNA) were shown to be present in sperm (Kramer and Krawetz, 1997; Wykes et al., 1997, 2000; Ostermeier et al., 2004; Miller et al., 2005; Rassoulzadegan et al., 2006; Krawetz et al., 2011; Yuan et al., 2016). Initially, the main classes of sncRNA found in sperm were miRNA, endo-siRNA and piRNA, the most abundant being piRNA (associated with repeated sequences Krawetz et al., 2011; Pantano et al., 2015; Hutcheon et al., 2017). MicroRNA (miRNA) and endogenous small interfering RNA (endo-siRNA) are sncRNA of about 20–24 nucleotides. They are synthetized in the nucleus and they maturate in the cytoplasm compartment. They are catalyzed by enzymes of the RNA interference machinery including DROSHA and DICER (Subramanyam and Blelloch, 2011; Yang and Lai, 2011) and participate to the RNA-induced silencing complex (RISC) involved in gene silencing (for review, see Martinez et al., 2002; Holoch and Moazed, 2015). Piwi-interacting RNA (termed piRNA) are another significant part of the sncRNA found in spermatozoa. They are DICER-independent and are 26–31 nucleotides long. As their name implies, piRNA interact with PIWI family proteins MIWI, MIWI2 and MILI (Malone and Hannon, 2009). piRNA are germ cell specific and comprise two subgroups according to their expression stage arbitrarily separated into pre-pachytene piRNA and pachytene piRNA (for review Luo et al., 2016). These piRNA are derived from repeated sequences and act on the silencing and DNA methylation of transposable elements (Deng and Lin, 2002; Kuramochi-Miyagawa et al., 2004, 2008; Aravin et al., 2007; Carmell et al., 2007; Luo et al., 2016).

Recent studies have discovered that small tRNA, a novel class of RNA, are in fact more abundant than miRNA as they constitute the majority of small ncRNA in sperm (Peng et al., 2012; Chen et al., 2016a; Sharma et al., 2016). The sperm small tRNA (stRNA) are mainly fragments of the 5′ end of tRNA, and range in size from 29 to 34 nt. Although details of their biogenesis remain unknown, they could have a potential role in transgenerational effects. This will be discussed below. Otherwise, sperm tRNA harbor numerous RNA modifications or RNA-editing that contribute to their stability such as 5-methylcytidine and N^2^-methylguanosine and has been recently summarized by Chen et al., (Chen et al., 2016b). Beside stRNA, 28s rRNA-derived small RNAs were also recently shown enriched in mature sperm and possibly associated with inflammatory situations (Chu et al., 2017).

Recent studies provide evidences that beside the spermatogenetic importance of sncRNA, there is a post-testicular transfer of sncRNA to sperm during post-testicular sperm maturation. It was shown that epididymosomes, a heterogeneous population of small membrane bound vesicles that are released from the epididymal epithelium (for review, see Sullivan and Saez, 2013) bring to transiting sperm cells a load of sncRNA (Reilly et al., 2016; Sharma et al., 2016). In line with these works, Dixon's group published recently that the content of sncRNA spermatozoa changed during the post-testicular maturation which takes place in the epididymis. The authors show that microRNAs (miRNAs) are highly represented in the spermatozoa of the proximal epididymis decreased during the epididymal transit, inversely the piRNA are enriched in the mature spermatozoa collected from cauda epididymis. These results demonstrate the complexity and dynamic nature of sncRNA profile of spermatozoa (Pantano et al., 2015; Hutcheon et al., 2017). To add to this complexity, a recent study, in which the miRNA profile of spermatozoa was investigated at the single cell level, suggests that spermatozoa from the same individual have different miRNA contents (Yu et al., 2017). Future studies are needed to shed light on the role of these post-testicular transferred sncRNA especially in the way they might influence embryo development, therefore contributing to another level of paternal inheritance (Figure 1).

## Contribution of the sperm epigenome to the embryo

### Paternal pronucleus

**Figure 2:**
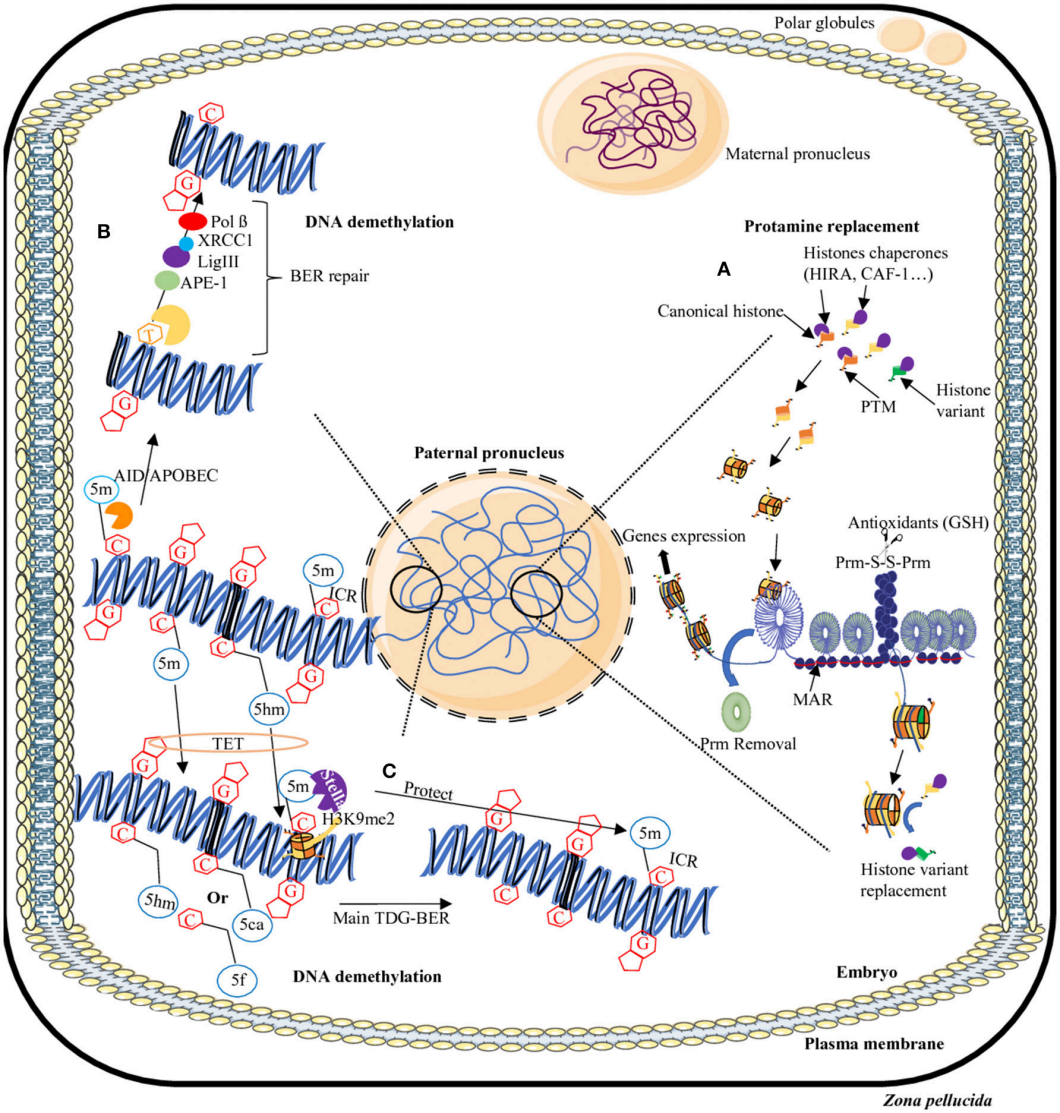
Dynamics of epigenome reprogramming of the male pronucleus. After fertilization, in one cell embryo, the sperm nucleus undergoes many remodeling events. First, protamines are replaced by maternally-derived histones **(A)**. In addition, in some areas of the sperm nucleus where persisting histones remain, some will be replaced by canonical or other variants. Furthermore, the majority of 5-methylcytosine residues (5 mC) will be erased by two processes: a TET-TDG-BER repair process that will introduce non-methylated cytosines **(C)** or the AID/APOBEC/BER pathway that will change mC in thymine (T) then will repair the T-G mismatches in non-methylated cytosines **(B)**. Some regions will be protected from DNA demethylation including repeats elements, transposons, and imprinting control regions (ICR) thanks to the presence of the Stella protein and H3K9me2 histone modification **(C)**. AID/APOBEC, cytidine deaminase; APE, Apurinic/Apyrimidic endonuclease; BER, Base excision repair; C, cytosine; G, guanine; GSH, Glutathione; 5m, 5-methylcytosine; 5hm, 5-hydroxymethylcytosine; 5f, 5-formylcytosine; 5caC, 5-carboxycytosine; Lig, Ligase; MAR, matrix attachment region; Pol, Polymerase; Prm, Protamine; PTM, post-translational modifications; S-S, disulfide bridge, TDG, Thymine DNA glycosylase; TET, Ten eleven translocation; XRCC1, X-ray repair cross-complementing protein 1.

As described above, the paternal nucleus is tightly compacted due to protamines. Upon fertilization, it is remodeled *via* the replacement of protamines with maternally derived histones (McLay and Clarke, 2003). The decondensed paternal DNA then expand to approximately three times the size of the mature sperm nucleus, resulting in the formation of the paternal pronucleus, compatible with DNA replication and the fusion of the maternal and paternal pronuclei. Its timing has been placed by many studies within the first hours after fertilization depending on the species concerned (Nonchev and Tsanev, 1990). In studies on human sperm, protamine removal was observed to be completed in the hour following intracytoplasmic sperm injection (Jones et al., 2011). In studies in which porcine sperm was used for *in vitro* fertilization (IVF), it was shown that 80% of the protamines were removed within 3 h post-fertilization (Shimada et al., 2000). On a mechanistic side, sperm nucleus protamine removal was shown to rely on an antioxidant activity mediated by maternal glutathione allowing for the reduction of disulfide bonds between protamines thus facilitating the paternal chromatin decondensation (Perreault et al., 1988; Figure 2).

Considering the fact that the sperm nucleus brings along persisting histones (canonical and variants) and associated PTMs, the question about the contribution of those persisting histones to the embryo was asked (i.e., are they replaced by maternal histones or not?). Of course, in view of the ongoing debate regarding the genomic location of sperm histones, their contribution and functional role in the embryo cannot be fully understood. Most data regarding the location of sperm histones after fecundation are based on immunodetection. It was reported that some sperm persisting histones variants are removed after fertilization. For example, H2AL1/2 rapidly disappear after fertilization in the mouse (Wu et al., 2008): First detected in the centromeres of spermatids, these variants remain enriched in heterochromatin domains until displaced from paternal DNA shortly after fertilization (Wu et al., 2008). In contrast, histone H3 replication-dependent variants H3.1 and H3.2 (Tagami et al., 2004) were detected in the male pronucleus after fertilization and prior to DNA synthesis, though in a much lower abundance than in maternal chromatin (van der Heijden et al., 2005, 2008; Hajkova et al., 2010). These sperm-derived proteins are detected until the zygotic S phase initiates, at which point they become indistinguishable from their newly incorporated maternal counterparts (van der Heijden et al., 2008). H3.3 plays an essential role during zygotic S-phase in the transcription of peri-centromeric domains that trigger their silencing following the first cell cycle by acquisition of H3K27 methylation in the male pronucleus (Santenard et al., 2010). Four hours after fertilization, the newly formed male pronucleus appears to carry only nucleosomes containing H3.3, while the female pronucleus exists in a relatively zero-H3.3 state (Santenard et al., 2010; Akiyama et al., 2011). Moreover, after fertilization, the embryo has to rearrange paternal pronucleus heterochromatin and euchromatin regions to form a functional embryonic genome (Probst et al., 2010; van de Werken et al., 2014). The pathway involved in this process is completely different between human and mouse embryos. In the human embryo, constitutive heterochromatin (cHC) of paternal pronucleus is established by the H3K9/HP1 maternal chromatin modifiers which recognize the canonical cHC (H3K9me3 and H4K20me3) carried by paternal genome. Whereas in the mouse embryo, the sperm paternal heterochromatin is devoid of canonical heterochromatin marks and is mainly established by PRC1/2 protein complexes (van der Heijden et al., 2006; Probst et al., 2010; Casanova et al., 2013; van de Werken et al., 2014).

Paternally-derived modified histones also seem to play a critical role in establishing a totipotent embryo. For a correct development, the paternal pronucleus is hyperacetylated shortly after fertilization with acetylation of lysines 5 and 16 of H4 and lysines 9, 14, 18, and 27 of histone H3 (Adenot et al., 1997; Santenard et al., 2010).

Beside remaining histones and protamines, the sperm nucleus contain Matrix Attachment Region (MAR) that allow the formation of the male pronucleus and the replication of the zygote DNA following fertilization (Linnemann et al., 2009). Indeed, intracytoplasmic injection of sperm-DNA alone without MAR into oocyte or co-injected with an isolated sperm nuclear matrix does not allow the formation of the male pronucleus or its replication. While injection of sperm nuclear matrix only associated with short DNA fragments at the base of the toroids (so DNA fragments associated with sperm MAR) allows the formation of the male pronucleus and DNA replication, despite sperm DNA degradation ranging from 20 to 50% (Shaman et al., 2007a,b).

### Paternal DNA demethylation/methylation

Before the fusion of the two pronuclei, a demethylation step takes place. The removal of the majority of 5mC in both genomes is crucial to establish the pluripotency of the inner cell mass of the blastocyst. For both the maternal and paternal pronuclei, DNA demethylation is mediated by (i) an active DNA demethylation process dependent on Tet enzymes, including Tet3, and the successive formation of 5 hmC, 5 caC, and 5 fC (Tahiliani et al., 2009). and (ii) a passive replication-dependent demethylation process (Guo et al., 2014; Shen et al., 2014; Wang et al., 2014). Most of the DNA inherited from the father appears to be demethylated before DNA replication (Mayer et al., 2000; Oswald et al., 2000; Santos et al., 2002). On the contrary, the maternal genome undergoes slower, mainly passive, replication-dependent DNA demethylation. Only a small but significant portion of its DNA appears to be actively demethylated by a Tet3-dependent process (Guo et al., 2014; Shen et al., 2014; Wang et al., 2014). Tet3 is predominantly located in the male pronucleus and results in an important accumulation of the 5 hmC mark in the male pronucleus (Gu et al., 2011; Iqbal et al., 2011; Amouroux et al., 2016). In zygotes deficient for *Tet3*, 5mC level remains constant on the paternal genome leading to a delay in the activation of paternal allele of genes essential for embryonic development, such as *Nanog* and *Oct4*. Interestingly, it is the maternally derived Tet3 enzyme which is responsible for the conversion of 5mC to 5 hmC in the paternal genome (Gu et al., 2011). A combined deficiency of *Tet1* and *Tet3* results in an increase in the 5mC mark and a loss of 5hmC at the eight-cell stage. In addition, these embryos show a decrease in the expression of genes involved in the biosynthesis of cholesterol; the few embryos that survived showed signs of a holoprosencephaly associated with neurological disorders (Kang et al., 2015).

The different methylated-cytosine derivatives (5hmC, 5fC, and 5caC) are ultimately transformed into non-methylated cytosine by the DNA repair pathway TDG-BER (standing for thymidine DNA glycosylase-base excision repair) that is independent of replication (Kohli and Zhang, 2013; Figure 2). TDG enzymes recognize only the 5fC and 5caC forms whereas 5hmC undergoes a deamination in 5hmU by the intervention of cytosine deaminase AID/APOBEC (Zhang et al., 2012; Xue et al., 2016). An abasic site is thus generated bringing in the players of the BER repair pathways including APE1 and XRCC1 (Weber et al., 2016). XRCC1 is located on the male pronucleus but absent from the female pronucleus (Hajkova et al., 2010; Wossidlo et al., 2010). In addition, the DNA methyltransferase DNMT1 is excluded from the nucleus of preimplantation embryos (Howell et al., 2001; Hirasawa et al., 2008), thus inducing the absence of re-methylation of the DNA immediately after fertilization.

Importantly, some regions of the paternal sperm nucleus will escape demethylation after fertilization, that is the case of some categories of transposable elements (IAP) and imprinted genes (Lane et al., 2003). Recently, studies have shown that a few single copy genes (which are not imprinted) also escape the demethylation wave (Hackett et al., 2013; Tang et al., 2015).

To explain the absence of active DNA demethylation in specific regions of the male pronucleus a mechanism has been evoked. It involves the Stella protein (also known as PGC7 or DPPA3) initially identified in the PGC. It is assumed that Stella protects from DNA demethylation, at particular paternal imprinted loci (such as *Rasgfr1*) by preventing the binding of the Tet3 protein and maintaining the presence of the histone methylation mark H3K9me2 (Nakamura et al., 2007, 2012; Bian and Yu, 2014). Interestingly, the persisting histone repressive marks are associated with imprinting repression such as H3K27me3, H3K9me3, and H4K20me3 (McEwen and Ferguson-Smith, 2010). The loss of function of the Stella protein leads to an arrest in embryo development associated with a loss of 5mC both in male and female pronuclei (Nakamura et al., 2007). Two other proteins have been identified to protect ICR from demethylation: Zfp57, a zinc finger protein of the KRAB family and Trim28. The interaction of these two proteins, which specifically target ICR, induces the recruitment of repressor complexes such as NuRD (Nucleosome Remodesling Deacetylase), Setdb1 (a histone methyltransferase) and DNMTs (Quenneville et al., 2011; Zuo et al., 2012). The loss of *Trim28* is embryonic lethal due in part to defects in the expression of imprinted genes (Messerschmidt et al., 2012) while the loss of *Setdb1* leads to a de-repression of retrotransposons and an increase in DNA double strand breaks (Kim et al., 2016).

### Paternally derived RNA

Since the discovery of sperm mRNA and non-coding RNA transmission to the embryo after fertilization, studies were conducted to determine the impact(s) and role(s) of these RNAs in embryonic development (Kramer and Krawetz, 1997; Ostermeier et al., 2004; Miller et al., 2005; Jodar et al., 2013). Regarding the sperm mRNA cargo, it was estimated that ~18,000 mRNA are delivered to the embryo (Ostermeier et al., 2004). The same group identified six of these sperm mRNA (mRNA for *clusterin, AKAP4, Prm2, Cdh13, Foxg1b*, and *Wnt5a*) and suspected them to participate to paternal pronucleus formation and to control the events of early embryo development. Regarding sperm-borne non-coding small RNA, studies have shown their impact in preimplantation embryo development. A striking example is that of miR-34c, a male germ line-specific miRNA that is transmitted to the embryo. Mir-34c was demonstrated to have a key role in the first division of the mouse embryo, as injection of a miR-34c inhibitor into zygotes was shown to induce an arrest in embryo cell division (Liu et al., 2012). This results from the inhibiting role of miR-34c on *Bcl2* (or B-cell leukemia/lymphoma 2) gene expression and on its anti-proliferative function.

It is worth noting that several studies have shown a link between DNA methylation and non-coding RNA. *Dnmt3A* and *3B* mRNA expression was shown to be negatively regulated by miR-29b in mouse early embryos and alterations of miR-29b activity was shown to change the DNA methylation level in mouse preimplantation embryos leading to developmental arrest at the morula stage (Zhang et al., 2015). In another study, when oocytes were fertilized using sperm derived from mice knocked-out for DICER or DROSHA, thus showing defective miRNAs/endo-siRNAs biogenesis, a deregulation in the expression of the embryo preimplantation genes was seen (Yuan et al., 2016). Interestingly, the phenotype of these embryos could be saved by injecting a pool of sperm RNA from WT mice (Yuan et al., 2016).

Altogether these studies show the importance of all the facets of sperm epigenome not only for sperm quality and fertilization abilities but also for subsequent embryo development. In addition, recent studies suggest that the sperm epigenome could go beyond that and have an impact on several generations.

## Transgenerational effects of the paternal epigenome

**Figure 3:**
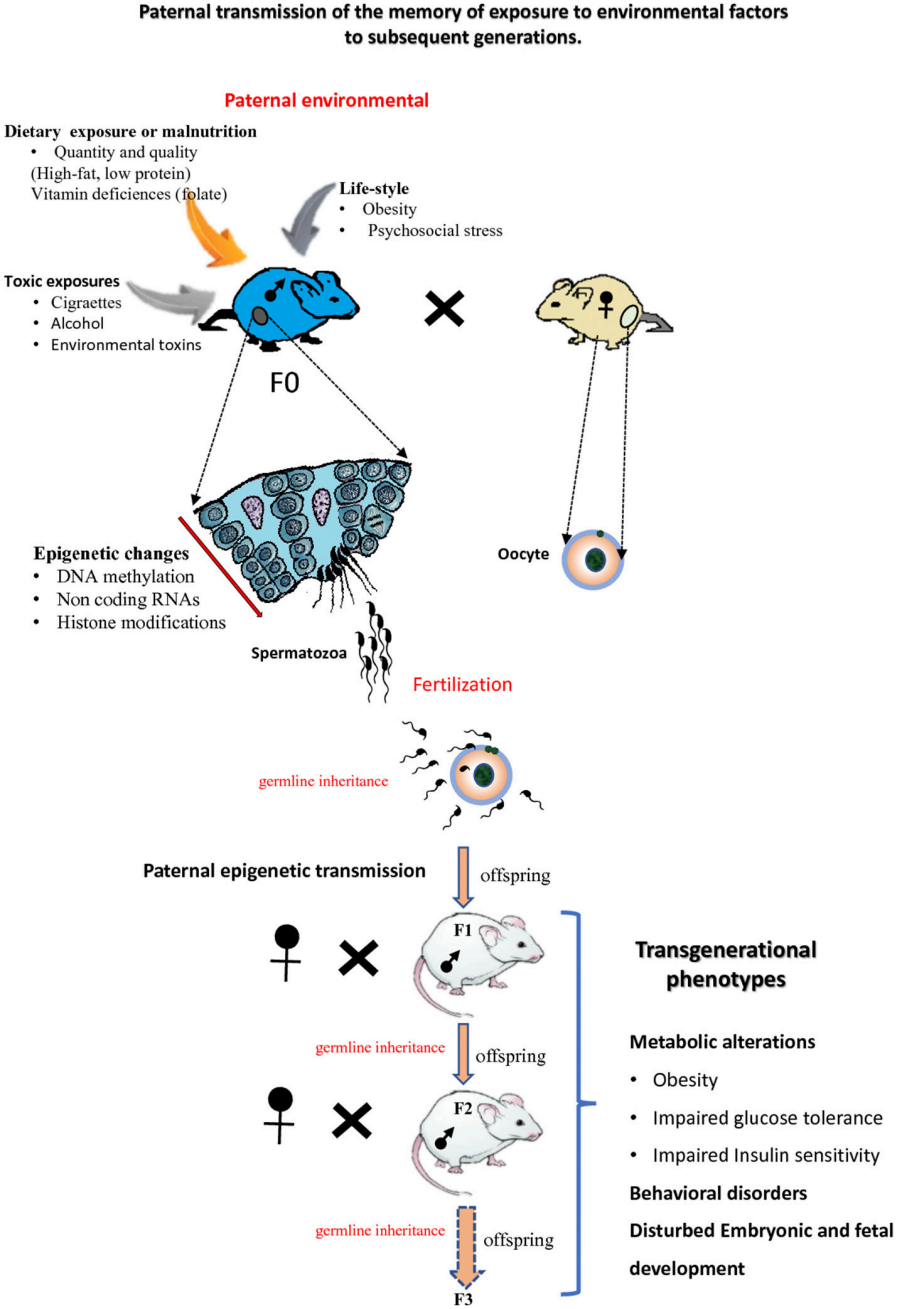
Paternal epigenetic transmission to subsequent generations. Environmental exposures of male mice (F0) can modify the sperm epigenome. Epigenetic information about these exposures can be transmitted to the next F1 generation and also to the F2 generation or more F3. The offspring of subsequent generations can be subject to transgenerational phenotypes.

Recent studies in animal models together with epidemiological data have suggested that epigenetic factors are responsible for the transmission of pathologies across generations. This phenomenon was called epigenetic inheritance or transgenerational epigenetic inheritance. Epigenetic inheritance involves the transmission of non-DNA base sequence information to the offspring over multiple generations *via* the germline. Epigenetic inheritance can occur when parents (F0 generation) are exposed to multiple environmental insults including nutritional stress, psychological stress, toxins and drugs exposure (Figure 3). Beside environmental origins, epigenetic inheritance might come from parents carrying mutations in epigenetic regulators (such as enzymes involved in the establishment of DNA methylation or chromatin modifications, etc.; Sharma et al., 2016). Both paternal and maternal gametes have been shown to transmit epigenetic information to the next generation (i.e., intergenerational transmission; Huypens et al., 2016). If epigenetic inheritance effects due to the mother lifestyle or/and environmental exposures particularly during oogenesis and during pregnancy (Gluckman et al., 2008; Jimenez-Chillaron et al., 2009; Radford et al., 2014) have been widely studied and are now well established, only recently has it become apparent that paternal epigenetics effects may also greatly influence offspring health (Schaefer and Nadeau, 2015). This part of the review aims at summarizing recent progresses made on paternal epigenetic inheritance and how environmental factors and paternal lifestyle can alter the paternal epigenetic information, in particular its DNA methylation/modification.

### Paternal experiences transmitted to the future generation via sperm epigenome

#### Examples of sperm epigenome modifications and consequences on the offspring

Several research groups using rodent models have recently shown that feeding male mice with unbalanced diets [either a low protein diet, a high-fat diet (HFD) or a Folate-deficient diet] could result in the appearance of metabolic disorders in the offspring that were directly linked to changes in sperm epigenetics (Carone et al., 2010; Ng et al., 2010; Fullston et al., 2013; Mejos et al., 2013; Wei J. et al., 2014; Schaefer and Nadeau, 2015; de Castro Barbosa et al., 2016; Schuster et al., 2016). The detected epigenetic alterations in the offspring concerned changes in DNA methylation at regulatory regions of genes involved in metabolic processes such as glucose–insulin homeostasis, cholesterol biosynthesis (Carone et al., 2010; Ng et al., 2010; Fullston et al., 2013).

For example, Ng et al. (2010) reported that feeding male mice with a HFD promoted in the female offspring a state of impaired glucose-insulin homeostasis. This was evidenced by alterations in the female offspring transcriptome of retroperitoneal adipose and pancreatic islet tissues. Changes in the methylation status of the female offspring *Il13ra2* gene that is part of the Jak–Stat signaling pathway was also detected in that model (Ng et al., 2010, 2014). In another study, it was reported that male mice consuming a low-protein diet produced offspring with increased level of cytosine methylation in *trans*-acting regulatory sequences of key genes involved in lipid and cholesterol biosynthetic pathways such as *Ppar*α (Carone et al., 2010). These changes were correlated with the down-regulation of the expression of the corresponding genes in the offspring (Carone et al., 2010). Interestingly, in that particular model, the authors did not show any significant change in cytosine methylation at classical imprinted loci in the offspring. In addition, the global cytosine methylation profile of the paternal sperm was not significantly different between male mice that were fed or not fed the diet. This suggested that paternal sperm DNA methylation modifications were probably not at the origin of the phenotypical trait recorded in the offspring in that particular model.

In different models, it was however shown that a diet-induced stress indeed provokes changes in the DNA methylation profile of the father's sperm cells (Fullston et al., 2013). In particular, Wei Y. et al. (2014) reported that a situation of diet-induced paternal prediabetes modifies the sperm methylation status of several genes such as the phosphatidylinositol (PI) 3-kinase subunits *Pik3ca and Pik3r1* of the insulin pathway. It was proposed that these altered sperm methylation profiles were at the origin of the metabolic disorders recorded in the next generations (Wei Y. et al., 2014).

In humans, the association between a paternal condition and epigenetic changes in their children has been described in many instances. For example, Soubry et al. explored putative correlations between paternal obesity and children epigenetic profiles (Soubry et al., 2013, 2015). They reported that children of obese fathers showed low methylation at differentially methylated regions (so-called DMRs) of several imprinted genes such as *Mest* and *Peg3*. Interestingly, a deregulation of the methylation status of these DMRs were reported elsewhere to be linked with the occurrence of chronic diseases and metabolic disorders in the offspring (Murphy and Jirtle, 2003; Jirtle and Skinner, 2007).

A great number of studies have demonstrated that environmental exposures play an important role in the accumulation of epigenetic patterns at specific loci, which further affects normal development and even causes various diseases via tightly regulated gene expression at the epigenetic level. In addition to the modifications of epigenome by nutrition, other environmental factors can alter epigenome such as toxins, unhealthy lifestyles, etc. (Schaefer and Nadeau, 2015). For example, in mice, male exposure to pesticides may increase the level of reactive oxygen species in testes, which has a severe impact on DNA methylation profiles in the sperm and this germline epigenetic information can be transmitted from generation to generations (Anway et al., 2006; Guerrero-Bosagna et al., 2012; Skinner et al., 2015). Moreover, the authors demonstrate that epigenetic mechanisms can influence and promote the occurrence of a number of DNA sequence mutations. Indeed, by analyzing the differential DNA methylation regions (epimutations) and genetic mutations (copy number variation: CNV), they demonstrate an increase in copy number variants associated with altered DNA methylation in the F3 generation (Skinner et al., 2015). Besides alterations in DNA methylation, exposure to environmental factors can lead to changes in sperm non-coding RNA and histone retention in sperm of DDT-exposed male mice (Skinner et al., 2018).

Since changes in the methylation status of the paternal genome were not always observed, other causative factors of paternal epigenetic inheritance have been suspected and looked for. Changes in chromatin packaging *via* sperm nuclear protein modifications and non-coding RNA content were brought forward and explored. As discussed above, sperm chromatin is mainly made of protamines which will be expelled shortly after fertilization; the so-called persisting histones, despite its limited amount, has therefore the most potential for a contribution to the next generation(s). Any modification of these persisting sperm histones may also be considered as part of the epigenetic information received by the future embryo. Very few studies have succeeded to establish a relation between sperm histone changes and transgenerational defects. In one particular study, Vassoler et al. (2013) showed that the administration of cocaine to male rats resulted in an increased level of brain-derived neurotrophic factor protein (BDNF) in the prefrontal cortex of their sons. This was shown to be associated with an increased level of acetylated histone H3 within the *Bdnf* promoter in the medial prefrontal cortex (Vassoler et al., 2013). This epigenetic modification was also found in the promoter of the *Bdnf* gene in the paternal sperm. Although interesting, these results are not sufficient to ascertain a transgenerational effect related to sperm histone modification because they did not show effect across several generations.

A more convincing association was shown *via* the use of a mutant mice for a protein involved in chromatin modification. In that model, mice overexpress a human version of histone H3 lysine 4 (H3K4) demethylase KDM1A during spermatogenesis. Spermatozoa of these mice showed reduced H3K4 dimethylation within CpG islands of genes implicated in development. In their progeny were observed severely impaired embryonic development and reduced survival rates across three generations (Siklenka et al., 2015). However, KDM1A was not found expressed in the germline of subsequent generations, the transgenerational defects were therefore unlikely to result from transmitted modifications of sperm histone PTM. Since no changes in DNA methylation were observed at CpG islands, the authors hypothesized that the transgenerational inheritance was mediated by sperm-borne RNA (Siklenka et al., 2015).

Several studies investigated specifically the role of small non-coding RNAs (sncRNAs) in transgenerational inheritance because they are long known to be present in the germ cell lineage and their involvement in the regulation of DNA methylation and histone modifications is well documented (Holoch and Moazed, 2015). Those studies found that sperm-derived ncRNAs, including miRNA and small tRNA derived from tRNAs, have the potential to influence embryonic development (see above) and lead to transgenerational inheritance (Holoch and Moazed, 2015; Rodgers et al., 2015; Chen et al., 2016a; Sharma et al., 2016). For example, a nutritional stress in rodents such as a high fat diet can modify the sperm miRNA content which constitutes potential epigenetic signals. These signals will drive offspring health and will initiate the transmission of metabolic abnormalities in future generations (Fullston et al., 2013). In 2016, two independent studies showed that a high-fat or a low-protein diet given to male mice was associated with increased levels of fragmented transfer RNAs species (stRNA) in sperm that were subsequently associated with metabolic disease in their offspring (Carone et al., 2010; Chen et al., 2016a; Sharma et al., 2016). To attest that sperm stRNA generated by the diets were at the origin of the inherited offspring phenotypes, both research groups microinjected these diet-induced small tRNA into control oocytes fertilized with sperm issued from males not subjected to the regimens. Chen et al. reported that a subset of stRNA, exhibits changes in expression profiles and RNA modifications in male mouse fed with HFD, compared with mice fed a normal diet (ND) male mouse. Injection of sperm stRNAs from HFD male mouse into normal zygotes generated metabolic disorders in the F1 offspring and altered gene expression of metabolic pathways in early embryos and islets of F1 offspring (Chen et al., 2016a) Moreover, the bioinformatics analyses show that sperm stRNA which are differentially expressed between HFD and ND match preferentially to gene promoters rather than coding regions which are associated with metabolic genes. Interestingly, Sharma et al, showed that other diet stresses such as protein restriction in male mice affects small RNA levels in mature sperm with increased level of 5′fragments of glycine tRNA. Furthermore, they showed that these stRNA are gained during the epididymal transit trough epididymosomes: vesicles that fuse with sperm during epididymal transit. The study showed that stRNA which are affected by low protein diet can regulate expression of transcripts driven by endogenous retroelements (MERVL) in the embryos (Carone et al., 2010; Sharma et al., 2016).

These 2016 studies confirmed an earlier report showing that male mice fed a western diet (high fat and high sugar diet) exhibited an altered sperm miRNA profile associated with an increase in metabolic disorders in their offspring (Grandjean et al., 2015). The microinjection of one of these differentially represented miRNAs, namely miR19, into fertilized eggs resulted in the appearance of similar phenotypes in the developed embryos (Grandjean et al., 2015).

Changes in sperm ncRNA content following an environmental stress is not limited to metabolic stresses since Gapp et al. (2014) reported that a traumatic stress such as maternal separation in early life was associated with behavioral and metabolic conditions in the progeny (Gapp et al., 2014). Here too, sperm miRNAs were shown to be involved since micro-injection of sperm miRNAs collected from traumatized males into fertilized oocytes led to similar phenotypes.

Altogether, these studies showed that the sperm epigenome in particular its DNA methylation profile or/and its ncRNA content has the potential to alter the health of the next generations.

## Conclusions and future prospects

The sperm epigenome, established during spermatogenesis in the testis, is highly specialized and unique. The studies mentioned in the present review demonstrate that sperm is more than a vehicle transferring its haploid genome to the oocyte. DNA modifications, chromatin proteins and associated marks as well as sperm-derived RNA (in particular sncRNA) constitute a specific epigenetic landscape, shown, in case of disruption, to result in male infertility, abnormal embryo development and/or transgenerational inheritance. Future studies will have to elucidate and clarify the role and underlying mechanisms of sperm epigenetics in those processes, in particular its impact on the offspring health. If one takes into account the additional effects due to variations in the oocyte epigenome, predicting the risk of diseases in the offspring associated with germ cell epigenome alterations will certainly be a rather complex issue.

## Author contributions

AC, JC, JH-B, and AK: drafted the manuscript; JD: critically reviewed the manuscript.

### Conflict of interest statement

The authors declare that the research was conducted in the absence of any commercial or financial relationships that could be construed as a potential conflict of interest.
